# The Roles of Epidemiologists, Laboratorians, and Public Health Agencies in Preventing Invasive *Cronobacter* Infection

**DOI:** 10.3389/fped.2015.00110

**Published:** 2015-12-24

**Authors:** Janine Jason

**Affiliations:** ^1^Jason and Jarvis Associates, LLC, Hilton Head Island, SC, USA

**Keywords:** *Cronobacter*, *sakazakii*, infant formula, neonatal infection, ST4

## Abstract

**Background:**

*Cronobacter* can cause severe, invasive infection in very young infants. These bacteria can also colonize or cause insignificant infections in immunocompromised, elderly, and/or hospitalized adults.

**Methods:**

This editorial review highlights key points addressed in the Frontiers Research Topic on *Cronobacter*, discusses the clinical presentation and epidemiology of *Cronobacter* infections, and examines the responses of public health agencies to this problem.

**Results:**

*Cronobacter* is rarely isolated from hospitalized, immunocompromised and/or elderly adults and does not cause significant disease in those patients. Certain species and strains, especially of *Cronobacter sakazakii*, can cause invasive illness in previously healthy infants <2 months of age. Multilocus sequence type 4 and clonal complex 4 (ST4/MLST 4) *C. sakazakii* are the predominant cause of *Cronobacter* meningitis, which occurs only in infants. These infections and this strain type are strongly linked to powdered infant formulas (PIF), which can also be contaminated with other *Cronobacter* strains. End-product testing is not intended to guarantee the absence of these organisms. WHO has made recommendations that can help decrease but will not eliminate the risk of this infection.

**Conclusion:**

To further define the spectrum of *Cronobacter*-associated disease, all isolates should be genetically typed using every currently available method, typing results should be linked to the associated epidemiologic and clinical data, and these data should be analyzed in a scientifically sound manner. Based on currently available information, more can be done now to prevent cause invasive infection in young infants. This includes encouragement of exclusive breastfeeding and/or use of commercially sterile ready-to-feed formulas in the first 2 months of life.

## Background

*Cronobacter* are Gram-negative, non-spore-forming, enteric coliform bacteria defined as a new *Enterobacter* species in 1980 by Farmer et al. (1). Recently, polyphasic taxonomic analysis has determined that this group of organisms consists of several genomospecies, so these organisms have been reclassified as novel species and subspecies within a novel genus, *Cronobacter* gen. *nov*. (2). *Cronobacter* isolates vary in regard to enterotoxin production, virulence, and thermotolerance. These variations – and *Cronobacter*’s taxonomy, ecology, reservoirs in nature, and characteristics in general – are almost certainly of clinical importance but have not been adequately studied and are not fully understood. Of the strains examined, many are extremely heat tolerant and can survive for long periods of time in a dry state [e.g., Ref. (3, 4)]. At least some form biofilms and thus resist the effectiveness of disinfectants (3–5). Lag times and microbiologic incubation periods for some *Cronobacter* strains can be as low as a matter of hours (6–8). In one study, an initial concentration of only 1 colony forming unit (CFU)/ml of *Enterobacter sakazakii* in reconstituted PIF grew to 10,000,000 in 10 h, at room temperature (9). Of note, growth would be even faster at body temperature.

*Cronobacter* are rarely isolated from clinical specimens and, when isolated, are largely from the very young or the elderly. Some strains can be highly pathogenic in neonates, especially infants who are premature or otherwise immunocompromised. *Cronobacter* occasionally colonize immunocompromised and/or elderly adults who are infected with other, significant pathogens. In these adults, *Cronobacter* rarely, if ever, cause significant symptomatic disease. In neonates, some strains of *Cronobacter* (and, in particular, some strains of *C. sakazakii*) cause invasive infection, even though the initial isolate is often from the gastrointestinal tract. The presentations and outcomes of neonatal *C. sakazakii* infection include seizures, hydrocephalus, developmental delay, and death.

A number of gastrointestinal and immunologic characteristics can contribute to an infant’s susceptibility to invasive *C. sakazakii* infection. First, the stomach environment of newborns, especially of premature babies, is less acidic than that of adults. Gastric and pancreatic secretions, mucus, and inherent enzymes are not produced at the levels found in an adult’s gastrointestinal tract. This would more readily permit any *C. sakazakii* entering the body through the mouth to survive in a viable state as it moves from the infant’s stomach to his intestines. Second, although enteric neuronal development is generally completed at a gestational age of 32 weeks (10), ineffective, uncoordinated, erratic, or static gut motility (peristalsis) can persist at least intermittently in even full-term infants. Peristalsis is an important mechanism for moving pathogenic organisms through and out of the gut, so dysfunctional movement may be a factor in *C. sakazakii* breaching the intestinal wall. Third, a fetus’s gut is sterile but this changes with passage through the maternal birth canal and first feeding. A newborn infant’s gastrointestinal system remains functionally inert until it is activated by food intake and microbial colonization. This means that until feeding is stably established, the feedback system between an infant’s gut and gut organisms does not function properly. Until then, an infant’s gut has suboptimal bacterial-epithelial “cross-talk,” i.e., a relative inability to differentiate pathogens from symbiotic organisms and to deal with each appropriately (11). Fourth, even if *C. sakazakii* is recognized as an enemy, an infant’s relatively permeable mucosa make him less able to retain the organism in his gastrointestinal system and remove it from his body. Last and perhaps most important, gut immunity develops gradually between fetal life and adulthood. Premature newborns, full-term newborns (born at 37–42 weeks gestation), neonates (<28 days old), and older infants (more than 28 days but <1 year old) differ significantly from one another and from older children and adults in regard to adequacy, strength, pattern, components, and rapidity of their immune response (11–13). Neutrophils can disrupt the intestinal tight junctions by releasing excessive interleukin (IL)-8 (12). Innate immunity, as represented by Natural Killer Cell (NK) production of interferon (IFN)-γ, dominates in early life (13).

The reservoirs for *Cronobacter species* are unknown. The majority of *E. sakazakii* and *C. sakazakii* isolates have come from infants, powdered infant formulas (PIFs), and factories producing milk powder (14). *Cronobacter species* have also been isolated from a number of other food substances [e.g., Ref. (4, 15)], food factories, and environments, including households (16); however, to my knowledge, no non-formula source of *E. sakazakii* or *C. sakazakii* has yet been causally linked to a case of invasive infection, although other sources have been examined in epidemiologic investigations.

## The Epidemiology of *Cronobacter* Infection

In Dr. Farmer’s first-hand review of *Cronobacter*’s microbiologic history (17), he appropriately credits Drs. Urmenyi and Franklin with the first clinical descriptions of invasive *E. sakazakii* disease, a description provided in a 1961 report of two 1958 cases of “neonatal death from pigmented coliform infection” (18). Those infants were born within months of each other, at a single United Kingdom hospital. Both developed hemorrhagic meningitis and died within 2 days of one another. One was full term, of normal birthweight, and had been discharged home. The other was premature, had low birthweight, and was still in the birth hospital when his symptoms began. Post mortem cultures from both infants grew identical “*pigmented cloaca*.” The infants had no known contact with one another and had not been on the same ward or nursery (18). In 1965, Jøker et al. reported a similar case, this time in Denmark. Dr. Farmer’s review article in this Frontiers Topic symposium describes how, over the following two decades, those isolates and similar pigmented coliforms were characterized, differentiated from other *Enterobacter*, and, in 1980, officially named *Enterobacter sakazakii*, according to the rules of the *Bacteriological Code* (17).

In the subsequent two decades, *Cronobacter* microbiologic research progressed but epidemiologic studies came to the forefront, in the form of outbreak investigations of pediatric *E. sakazakii* infections in the Netherlands (1983), Greece (1987), Iceland (1989), the United States [U.S.] (1989 and 2001), Belgium (2001), Israel (2001), France (2004), and New Zealand (2004) (19–21). In the early investigations, no source was identified. Tellingly, nutrition was not examined in those outbreaks. The investigations of subsequent outbreaks repeatedly documented a strong statistical and microbiological link between PIF and invasive *E. sakazakii* infection. The outbreak studies were increasingly elegant and thorough. In all 10 outbreaks, at least some environmental testing had been done. In the eight outbreaks in which nutrition was examined, all the infected infants had received some type of PIF and there was a statistically powerful association between *E. sakazakii* infection and consumption of a specific PIF. In five outbreaks, *E. sakazakii* was isolated from PIF but not from any environmental samples; in two, *E. sakazakii* was isolated from PIF and the blender used in blending that specific PIF; and in one, *E. sakazakii* was isolated from PIF, a dish brush, and a stirring spoon. Four outbreak reports specified that previously unopened containers of PIF were cultured; of these, three were positive. In seven of the eight outbreaks where *E. sakazakii* was typed, a PIF isolate was indistinguishable from patient isolate(s), based on the results from one or another of the typing techniques summarized in Dr. Yan’s concise article in this Frontiers symposium (22). The formula consumed by infected infants in each of these outbreaks yielded *E. sakazakii*; in two outbreaks, formula from previously unopened cans from the same manufacturing batch also yielded *E. sakazakii*. In three outbreaks, investigators were able to show both statistical and microbiological associations between *E. sakazakii* infection and the consumption of PIF (23–25). In these investigations, there was no evidence of infant-to-infant or environmental transmission; all the infected infants had consumed the implicated formula (23–25).

At the time these investigations were done, epidemiologists had reason to suspect that *E. sakazakii* infections might be caused by a nutritional substance. *Entero* is derived from the Greek word for intestines. *Enterobacter* got that name because they have long been known to enter the body through the digestive system and reside in the intestines. There was also good reason to consider PIF as the potential nutritional source of these *E. sakazakii* infections. Infants are fed PIF and PIF is not sterile. Studies had already shown that strains of *E. sakazakii* could contaminate PIF components, become endemic in the post-pasteurization dry-processing areas of PIF processing plants, survive the dry-processing procedures, survive in a dry state for long periods of time, and become biologically active in the presence of moisture. Thus, it did not require great insight to include PIF as one of the independent variables studied in these outbreaks. However, these studies do demonstrate the elegant effectiveness of interactively applying epidemiologic, statistics, and microbiologic scientific techniques to outbreaks involving extremely few cases.

Concurrent with the above-described epidemiologic investigations, microbiologists and food scientists investigated the extent and nature of PIF contamination with *E. sakazakii*. Muytjens et al. examined 141 different powdered formulas on the market produced in 35 countries and found *E. sakazakii* and other *Enterobacteriaceae* were common contaminants (26). He isolated *E. sakazakii* at levels ranging from 0.36 to 66 CFU/100 g from 20 formula samples from 13 countries (27), even though all those formulas met the contemporaneous microbiological specifications for coliform counts in PIF (<3 CFU/g). A Canadian survey that investigated the incidence of *E. sakazakii* in PIF isolated the organism from eight of 120 cans on the market, from five different manufacturers (28).

In 2002, a Belgian infant who received PIF died from an *E. sakazakii* infection. The manufacturer retested the implicated batch (also referred to as a “lot”) and, although the prerelease testing of the product had been negative for *E. sakazakii*, the additional testing was positive. The company voluntarily recalled the product. Following the epidemiologic investigation of a 2001 U.S. newborn intensive care unit outbreak (23), a lot (also referred to as a “batch”) of the implicated product was recalled and the U.S. Food and Drug Administration (FDA) did a field survey of 10 U.S. powdered formula manufacturing plants run by various manufacturers (29). In that field survey, which included 22 finished product samples, five (22.7%) had a most probable number (MPN) above what was then the currently acceptable detection limit (0.003 MPN per gram of PIF). The positive samples included four of 14 (28.5%) formulas for full-term and one of four (25%) formulas for pre-term infants. The results were not related to product type (milk vs. soy) or manufacturing processing used (e.g., wet mixing-spray drying vs. dry blending) (29). Despite these findings, infant formula manufacturers did not readily accept that their products played any role in these infections or were of any risk to healthy, full-term infants (30). Public health and regulatory agencies did not fully agree with the manufacturers.

In 2002, the FDA released a protocol for isolation and enumeration of *E. sakazakii* from dehydrated PIF [formerly at http://www.fda.gov/Food/ScienceResearch/LaboratoryMethods/BacteriologicalAnalyticalManualBAM/ucm109656.htm. No longer available on at the FDA website.] The protocol was an improvement over formula companies’ standing protocols but had significant flaws and was worrisome in that it did not attempt to assure the absence of *Cronobacter* in end-product. Furthermore, the FDA did not require manufacturers to use the FDA protocol for PIF end-product testing, nor did the FDA address environmental testing.

Also in 2002, the FDA sent out a “*Letter to Health Care Professionals*,” informing them that PIF and powdered breast milk supplements are not sterile and warning that premature infants and infants with underlying medical conditions could become infected with *E. sakazakii* (31). In the letter, the FDA recommended PIF be avoided in newborn intensive care units unless there was no alternative.

Parents of newborn or even of newborn premature infants have never received similar information, although, even at that time (i.e., 2002–2003) invasive *E. sakazakii* infection had occurred in term and non-hospitalized infants (21). There is reason to believe infants’ caretakers were then and remain in need of this information: In a 2005–2006 nationally representative survey of U.S. mothers of 2-month-old infants, only 29.5% correctly answered that PIFs were “*likely to contain germs*,” while 31.1% incorrectly thought that commercially sterile, ready-to-feed formulas (RTFs) were likely to contain them (32). Of note, that survey was done after most, if not all, formula manufacturers had bowed to encouragement to educate the consumer by adding the statement “PIF is not sterile” to their package labels.

Based on the accumulating epidemiologic evidence and product-testing data, the World Health Organization (WHO) acknowledged there was a problem that needed to be addressed. In 2004–2008, the Food and Agriculture Organization of the United Nations (FAO) and World Health Organization (WHO) held a series of advisory meetings concerning *E. sakazakii* (33, 34). These resulted in a risk assessment model, as well as a series of reports and recommendations [FAO/WHO^1^]. In this Frontiers Topic symposium, Dr. Parra Flores applies the WHO’s risk assessment tool to explore the effect of temperature on *Cronobacter* growth (35). In 2006, WHO stated that “*contaminated PIF has been convincingly shown, both epidemiologically and microbiologically, to be the vehicle and source of infection in infants*” (36). However, WHO’s guidelines and model focus on decreasing the risk associated with contaminated PIF, not preventing the contamination.

Even as WHO was formulating a response to the *Cronobacter* problem, reports of intrinsic PIF contamination continued to be made to the European Rapid Alert System for Food and Feed (RASFF). Between 2002 and 2008, eleven lots of PIF were cleared and released for sale by their manufacturers, distributed in Europe, and subsequently reported by outside authorities to be contaminated with *E. sakazakii* (37). *E. sakazakii* infections and colonizations were reportedly associated with at least three of these contaminated products (37). At least one other lot was reported to RASFF, by France in 2009^2^. Of note, the U.S. did not and does not have a reporting system for product contamination or infections with this organism. They are currently reportable in only one State – Minnesota (personal communication with Dr. A. Bowen, an epidemiologist at CDC, January 2012).

At the beginning of the twenty-first century, while health and regulatory agencies were developing models and guidelines, laboratorians were making striking advances. Their research followed two avenues: (a) typing and characterization of isolates and (b) investigating, characterizing, and statistically evaluating PIF contamination and end-product testing. These topics are addressed in this symposium’s articles by Dr. Yan and Dr. Kalyantanda (22, 38) but, in the following paragraphs, this editorial review will briefly discuss what are arguably the two most significant developments to date: identification of highly stable *C. sakazakii* clone with a high propensity for neonatal meningitis and determination that pre-market PIF testing cannot assure an absence of clinically significant contamination.

As noted in Dr. Farmer’s review article in this Frontiers Topic symposium (17), in 2007–2008, Iverson et al. proposed that *Cronobacter* be recognized as a new genus. That genus was to include organisms previously classified as *E. sakazakii* (2). As of this writing, 10 species and 3 subspecies of *Cronobacter* have been named and described. In their contribution to this symposium, Dr. Yan and Dr. Fanning succinctly describe the various and increasing number of laboratory techniques for identifying, tracking, comparing, and characterizing *Cronobacter* isolates (22, 38). Also in this Frontiers Topics symposium, Dr. Tall presents a pan genomic DNA microarray platform he developed and has used to document and characterize the genomic diversity among each member of the genus (39). That approach, as well as whole genome sequencing, will be powerful tools for genomic research on *Cronobacter* but one technique has already proved invaluable: a multi-locus sequence typing (MLST) scheme developed by Baldwin et al. (40). Application of that scheme to stored clinical, food, and environmental isolates has provided key information about the sources of various *Cronobacter* species and strains, the cause of *Cronobacter* meningitis, and the reasons why infants have severe clinical symptoms and adults do not (40–42).

Baldwin determined sequence types in relation to source and biotype, using 60 *C. sakazakii* and 16 *C. malonaticus* strains from clinical and non-clinical sources collected between 1951 and 2008 in the U.S., Canada, Europe, New Zealand, and the Far East. Twenty-two of 60 *C. sakazakii* isolates had sequence type 4 (ST4). Nine of those ST4 isolates were from clinical samples. Seven of the other 13 ST4 isolates were from infant formula (40). Joseph and Forsythe examined 41 *C. sakazakii* isolates, some of which appear to have been among those described by Baldwin (40, 41). Twenty of the 41 isolates were ST4; the remainder were one or another of nine other types. Of the 30 *C. sakazakii* isolates with known patient-source details, the only one from an adult patient was ST1, i.e., not ST4. Of the 20 ST4 strains, 10 were from neonates, seven from infants, one from a child, and two had no patient information. Although the seven housekeeping genes for MLST analysis are not virulence related, a large proportion of severe neonatal infections were caused by isolates with the ST4 sequence type: i.e., 9 of 12 isolates from meningitis cases were ST4 strains; the remaining ST4 strains were from a bacteremia case, necrotizing enterocolitis cases (two), and an undefined infection (one). The clinical disorders related to six of the ST4 strain isolates were unknown. Nine of 12 meningitis isolates were ST4. Joseph and Forsythe concluded that *C. sakazakii* ST4 and the related Clonal Complex 4 (CC4) represent a highly stable clone with a high propensity for neonatal meningitis (41, 42). They noted that *C. sakazakii* ST4 strains have been isolated from seven countries for >50 years and the earliest (1950) non-clinical isolate was from a can of dried milk. Joseph and Forsythe proposed that the relationships between genotypes and different age groups may reflect exposure to different genotypes of *C. sakazakii* according to age-related diet and lifestyle. In the context of Baldwin and Forsythe’s finding of a predominance of ST4 types in *C. sakazakii* isolates from infant formulas, Joseph and Forsythe’s results suggest that the key exposure in *C. sakazakii*-infected infants is intrinsically contaminated infant formula.

Of note, in an FDA investigation of 14 cases of community–acquired pediatric *Cronobacter* infections between May, 2010 and December, 2011, Dr. Tall et al. did ST typing. These isolates were not related to those previously studied by Dr. Baldwin et al. but they were also predominantly ST4 (43). MLST testing of isolates from numerous food and other sources from around the world is progressing at a rapid pace and indicates that MLST types vary by food and food-factory source, clinical source, and country. As noted by Dr. Kalyantanda in this Frontiers Topic symposium, results are being posted at an online database^3^ (38).

By far, the most significant development related to product safety and testing was a 2011 study, done in collaboration with a European branch a formula manufacturing company (44). In it, Jongenburger et al. examined a production “lot” (also referred to as a “batch”) of PIF. That batch had passed pre-market testing and been released to market, but it was subsequently found to be contaminated with *Cronobacter*. The researchers compared the contaminated batch to one recalled for non-microbiologic reasons and determined that: (a) both lots were actually positive for *Cronobacter* but a greater number of samples from the contaminated lot were positive, (b) the contamination was not homogeneous, and (c) the recalled lot had higher levels of contamination (44). The estimated degree of contamination varied among the positive samples and included clusters of 3–560 cells. The two largest clusters, of 123 and 560 cells, originated from two product bags only, consistent with clusters being present in a limited number of servings. Thus, an individual infant can be exposed to a significant inoculum, even if the remaining product in that can, the remaining product in that lot, and the product from that lot fed to other infants are free of *Cronobacter*. Dr. Jongenburger concluded that “*finding such clusters is like looking for a needle in a haystack*” and “*when these clusters end up in one or a limited number of servings of an individual consumer, they may significantly impact public health*” (44).

Dr. Jongenburger’s findings are especially meaningful because the sparse research on *Cronobacter*’s infectious dose suggests it can quite low, i.e., on the order of 1000 cells (3, 45) and quite possibly even lower for virulent strains. Extensive research has been done on related parameters, including lag time, generation time, and growth rate. Unfortunately, this research has not yet been done using specific isolates associated with proven, invasive clinical infections, e.g., *C. sakazakii* ST4 or CC4 isolates associated with *Cronobacter* meningitis in young infants. However, using the data that are available, albeit for non-virulent isolates, we can get a sense of how quickly an infectious dose of 1000 cells can be reached when contamination is clustered or clumped. Let us take a relatively small cluster of 10 cells. (Of note, in end-product testing, that cluster would appear as one CFU, even though all 10 cells could multiply independently.) Let us be conservative and round up the generation time determined by Dr. Parra Flores in his article in this Frontiers Topic symposium (35), which is consistent with the value found by others. Using a generation time of a half hour at 35°C (a temperature roughly comparable to the 36–37°C body temperature of an afebrile neonate) and assuming virulent *Cronobacter* strains thrive in the neonatal gastrointestinal tract, the 10-cell cluster would reach an infectious dose of 1,000 CFU within 3.5 h, a time interval consistent with the time it takes an infant to consume and digest a formula feeding.

To summarize, by the end of 2011, it had been determined that *Cronobacter* meningitis is associated with ST4 strains of *C. sakazakii*, these strains are found widely throughout PIF and PIF production facilities, and they appear to be stable clones present for many years in many countries. Other *Cronobacter* can be found in PIF and PIF factories but the evidence thus far suggests they do not cause meningitis. Dry-product *Cronobacter* contamination can be non-homogeneous, clustered, and clumped. End-product testing is inadequate for preventing that contamination and isolated contamination could be present even if the batch were negative to end-product testing and every other can in the batch was negative.

This laboratory progress was scientifically significant but, in late 2011, epidemiology again came to the forefront – if only in regard to the public media. Single reports of *Cronobacter* illness in infants in Missouri and Illinois caused the U.S. Centers for Disease Control and Prevention (CDC) to ask public health officials around the country to look for other cases of *Cronobacter* infection among infants. This generated reports of two additional cases, one in Oklahoma and one in Florida, bringing the 2011 U.S. case total to 13 (46). According to CDC’s *Cronobacter* website, CDC investigated the four cases occurring after November 1, 2011. All had been fed PIF, three had meningitis, and two died. The website indicates that CDC could not determine a source and DNA fingerprinting of isolates from two or the cases suggested they were unrelated. ST testing is not mentioned, nor if any of these cases were among those tested by Dr. Tall and found to be predominantly ST4, the strain type associated with both meningitis and PIF (see above) (43). The CDC website indicates that factory-testing was negative and notes that “*Cronobacter bacteria are found in the environment and in hospitals and homes*.” These statements ignore the wide variety in *Cronobacter* species and strains, the ST studies, and the extensive epidemiologic evidence supporting that PIF is the primary source of the specific ST type causing most cases of invasive *Cronobacter* meningitis.

At the time these 2011 cases were reported to the general public, I had reviewed the *Cronobacter* literature and material from formula manufacturers, as well as CDC, FDA, and WHO records and documents up to and through 2010, as well as the medical records of a number of *Cronobacter* cases that had not been investigated by those agencies. I was concerned that statements made in relation to these cases did not seem to match the material I had in hand. I therefore used that material to perform three epidemiologic analytic studies related to invasive *E. sakazakii/Cronobacter* infection. These studies were published as an article in *Pediatrics*, the peer-reviewed journal of the American Academy of Pediatrics (21). In this Frontiers editorial review i will now briefly describe those three epidemiologic studies and discuss how the findings and the current state of *Cronobacter* science are discordant with CDC’s current approach to epidemiologic surveillance and investigations, as well as with the FDA’s current PIF and PIF factory surveillance and testing protocols.

Two of these epidemiologic studies examined data on all reported cases of pediatric *E. sakazakii/Cronobacter* infection occurring worldwide from 1958 through 2010. The two studies also analyzed in greater depth all cases of invasive infection occurring in children who appeared to have been previously healthy, i.e., without any known underlying immunodeficiency or disorder. “Invasive disease” was defined as necrotizing enterocolitis, urinary tract infection, bacteremia, and/or meningitis, since this definition had been used in the previous *Cronobacter* literature [e.g., Ref. (19, 20)]. My first study was an epidemiologic characterization of all reported cases of invasive *E. sakazakii* or *Cronobacter* infection occurring in previously healthy children in 1958–2010. The second was a statistical comparison of cases occurring in 1958–2003 to those occurring in 2004–2010, i.e., before and after the dissemination of the FDA’s “*2002 Letter/Revised Letter to Health Care Professionals*,” in which the FDA recommended that “*PIFs not be used in neonatal intensive care settings unless there is no alternative available*” (31). As noted above, the FDA did not provide any guidance or recommendations to caretakers of infants living at home, even though cases had occurred in home settings by the time of the FDA’s *Letter to Healthcare Professionals*. The third study was a cross-sectional cost comparison of milk-based and soy-based: (a) PIF, (b) commercially sterile RTF, and (c) commercially sterile concentrates (which are to be diluted one-to-one with water prior to feeding). This study included data on a variety of brands of formula. It was done in response to statements made by the FDA, manufacturers, and others, in which they acknowledge that properly manufactured RTF cannot infect infants but insist that using RTF is not an option because it is significantly more expensive than PIF. These spokespersons provide only anecdotal statements and outdated references for their opinion [see, for example, Ref. (47)]. Therefore, my third study was intended to provide substantive, current data rather than anecdotes.

**Figure 1:**
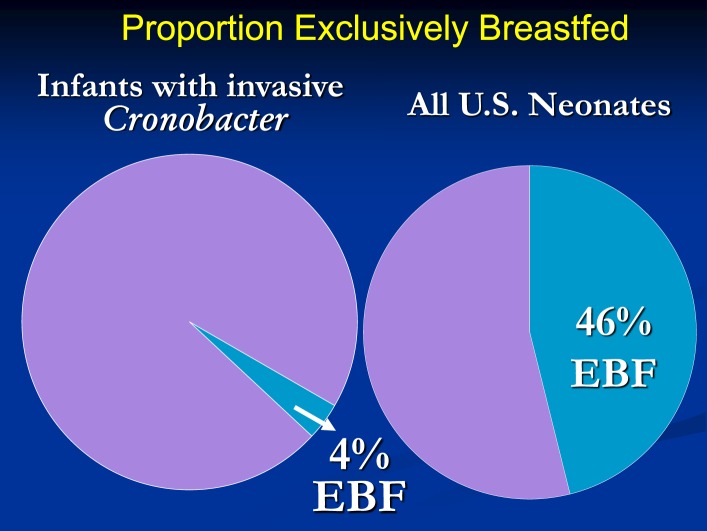
**Proportion of infants with invasive *Cronobacter* infection and proportion of all U.S. neonates who are exclusively breastfed**.

There were several striking findings in the first analytic study. First, for the entire time period examined, only one previously healthy infant who became invasively infected was older than 2 months of age at the time of symptom onset. Second, the majority of infected infants had meningitis and a third had documented bacteremia (Table 1). Third, 90% of invasively infected infants had received PIF at some point in time prior to onset of symptoms; this did not differ significantly between the two periods (Table 2). Only 4% of infants with invasive *Cronobacter* infection, worldwide, had been exclusively breastfed (EBF) (Figure 1). None of these breastfed infants resided in the U.S. but, for comparison: 46% of all U.S. neonates are EBF (48). Thus the proportion breastfed is much lower than would be expected, even if all the EBF infants had lived in the U.S.

**Table 1:** **Age and diagnoses of all reported infants without underlying disorders, invasively infected with *Cronobacter*, by time period**.

	1958–2003	2004–2010	Total	*P* value^a^
**Age at onset of symptoms**				
≤30 days/1 month old	53/66 (80%)	27/30 (90%)	80/96 (83%)	NS
≤60 days/2 months old	65/66 (98%)	30/30 (100%)	95/96 (99%)	NS
**Diagnoses^b^**				
Meningitis	38/68 (56%)	22/30 (73%)	60/98 (61%)	NS
Bacteremia	21/68 (31%)	14/30 (47%)	35/98 (36%)	NS
NEC	22/68 (32%)	1/30 (3%)	23/98 (23%)	*P* = 0.001
UTI	1/68 (2%)	0/30 (0%)	1/98 (1%)	NS

*^a^Fisher’s exact tests and Freeman–Halton extension of the Fisher’s exact probability test for a 2 × 3 table. Not significant (NS) if *P* ≥ 0.05. Totals percents may not equal 100 due to rounding*.

*^b^Some patients had more than one diagnosis. Specifically, 18 patients with meningitis also had proven bacteremia and 2 also had necrotizing enterocolitis (NEC). One patient with bacteremia also had NEC and one also had a urinary tract infection (UTI). *P* values are for proportion with each individual diagnosis*.

**Table 2:** **Number and proportion of reported infants without underlying disorders, invasively infected with *Cronobacter*, by time period and nutrition source**.

Nutrition source^a^^,^^b^^,^^c^	1958–2003	2004–2010	Total	*P* value^d^
Any PIF or HMF	51/54 (94%)	25/30 (83%)	76/84 (90%)	NS
Any breast milk	10/54 (18%)	12/29 (41%)	22/83 (26%)	*P* = 0.036
Any RTF	6/53 (9%)	13/29 (45%)	19/82 (23%)	*P* = 0.003
Any concentrate	1/53 (2%)	2/29 (7%)	3/82 (4%)	NS

*^a^Documented nutrition at any time prior to onset of symptoms, based on the best available information, including from medical records, CDC files, parent report, publications, and communications with publication authors. PIF, powdered infant formula; HMF, powdered human milk fortifier. Total percents may not equal 100 due to rounding. Denominators include only those for whom data were known*.

^b^This includes one infant who received formula that was likely but not definitely PIF and definitely did not receive breast milk (2000 case, personal communication, J. Burdette, October 6, 2011). This infant is included in the denominator for “any breast milk” and not in any numerators. The category also includes an infant who definitely received a recalled, contaminated lot of PIF but I could not determine if he received breast milk or other formulas as well (Belgium 2002). This infant is included in the numerator and denominator for “any PIF.” A third infant in this category is a term newborn recorded on a CDC line list as not having received PIF but without information concerning what, if any, enteral feeding she did receive (AZ 2009). This infant is included in the denominator of “Any PIF or HMF” and is not included in “Any breast milk,” “Any RTF,” and “Any concentrate.”

*^c^Categories are not mutually exclusive; therefore, total percent is >100. Numbers are for those who had the specified nutrition noted*.

*^d^Fisher’s exact tests. Not considered significant (NS) if *P* ≥ 0.05*.

The second set of analyses documented several significant differences between cases occurring in 2004–2010 compared to 1958–2003. The proportion with necrotizing enterocolitis, a disease that occurs in hospitalized premature infants, was significantly lower in 2004–2010 (Table 1). This decrease is consistent with the following findings: in 2004–2010, a significantly higher proportion of invasively infected infants were full term, normal birthweight, and living at home when symptoms began (Table 3). Also, in 2004–2010, a significantly higher proportion of infected infants had been given multiple types of nutrition prior to onset of symptoms (Table 2). This was largely due to their receiving RTF in their birth hospital and being switched to PIF at home (data not shown). This combination of findings supports that invasive infections can occur in healthy newborns and the proportion of cases in this group increased in the years following the FDA recommendation to avoid feeding PIF to hospitalized infants.

**Table 3:** **Characteristics of all reported infants without underlying disorders, invasively infected with *Cronobacter*, by time period**.

Characteristic^a^	1958–2003	2004–2010	Total	*P* value^b^
Premature	48/63 (76%)	12/29 (41%)	60/92 (65%)	
Full term	15/63 (24%)	17/29 (59%)	32/92 (35%)	*P* = 0.002
BW <2500 g	44/55 (80%)	10/24 (42%)	54/79 (68%)	
BW ≥ 2500 g	11/55 (20%)	14/24 (58%)	25/79 (32%)	*P* = 0.001
Place of symptom onset:				
Hospital	48/61 (79%)	14/29 (48%)^c^	62/90 (69%)	
Home	13/61 (21%)	15/29 (52%)	28/90 (31%)	*P* = 0.007

*^a^An infant was considered full term if the records indicated that was the case and/or the gestational age was specified as being at least 37 weeks. An infant was considered premature if the records indicated that was the case and/or the gestational age was less than 37 weeks. BW, birthweight. Table excludes patients for whom the specified data are unknown; there were a total of 68 infants in 1958–2003 and 30 in 2004–2010*.

*^b^Fisher’s exact tests and Freeman-Halton extension of the Fisher’s exact probability test for a 2 × 3 table. Not significant (NS) if *P* ≥ 0.05. Totals percents may not equal 100 due to rounding*.

*^c^This category includes one infant who became ill 12 h after leaving the hospital and another who was noted to be ill on the day of hospital discharge and was reportedly symptomatic while in the hospital*.

The third study addressed the relative costs two alternative forms of infant formula, RTF and concentrates, compared to PIF. A cross-sectional survey was done in September 2011. It compared on-line-prices for six formulas commonly used from birth to 6 or 12 months of age and available in PIF, RTF, and concentrate formulations. Prices varied relatively widely within and among brands, products, formulations, and stores. The approximate daily (four ounces of formula every 4 h) costs for feeding a neonate the least expensive formula of each type were calculated and compared. Details are provided in the footnotes to Table 4. The results support that if an infant’s caretaker was not brand-committed, the cost of RTF did not differ meaningfully from the cost of PIF. Specifically, milk-based RTF costs only 84 cents more a day than milk-based PIF and soy-based RTF costs 24 cents less a day than soy-based PIF (Table 4).

**Table 4:** **Per ounce price and price difference, by type and form of infant formula, comparing least-expensive available product in stated category^a^^,^^b^**.

	Actual cost/ounce^c^	Actual cost differences compared to powdered	
Milk-based formula:			
Powdered	0.121	NA^d^	NA
Ready-to-Feed	0.156	29%	0.035
Concentrate	0.137	13%	0.016
Soy-based formula:			
Powdered	0.140	NA	NA
Ready-to-Feed	0.130	-7%	-0.010
Concentrate	0.140	0%	0

*^a^Costs were determined for six formulas available for neonates and young infants (and for use by a premature or immunocompromised infant as/if recommended by that infant’s pediatrician): Enfamil (milk-based) (five stores for PIF and RTF, two stores for concentrate); ProSobee LIPIL (soy-based) (five stores for PIF, three stores for RTF, and two stores for concentrate); good start with iron, gentle or gentle plus (milk-based) (*n* = 5 stores for PIF, four stores for RTF, and three stores for concentrate); Good Start soy, Supreme or Supreme Plus (four stores for PIF, three stores for RTF, and two stores for concentrate); Similac Advance (milk-based) (five stores for PIF, RTF, and concentrate); and Isomil (soy-based) (five stores for PIF, four for RTF and concentrate). Prices were obtained in September 2011, for the least expensive packaging options, from the following internet sites: Amazon.com, Babies-R-Us, CVS, Diapers.com, and Walmart. Not all sites carried all brands of each product but all sites carried at least one brand each of a powdered, ready-to-feed, and concentrate product. Price ranges are for any of the assessed brands at any of the assessed internet sites*.

*^b^Lowest priced product of any brand, at any store. Numbers reflect actual costs and cost differences for those products*.

*^c^In dollars per fluid ounce of prepared formula*.

*^d^Non-applicable (NA)*.

The following consumer recommendation appeared on CDC’s *Cronobacter* webpage in December 2012: “*If your baby gets formula, choose infant formula sold in liquid form, especially when your baby is a newborn or very young*”^4^. Unfortunately, CDC’s one epidemiologic contribution to the recent *Cronobacter* literature, a 2014 article by Patrick et al. published in CDC’s journal *Emerging Infectious Diseases*, not as insightful (49). It is seriously flawed and misleading in a number of respects but this editorial review will focus on a few key issues.

The 2014 Patrick/CDC study examined data collected through CDC’s FoodNet Program [Foodborne Diseases Active Surveillance Network (FoodNet)] (50), an excellent laboratory isolate surveillance program but one that does not collect detailed patient information. The FoodNet data support what was previously known: with rare exception, clinical *Cronobacter* isolates come from the very young and the very old. Despite that bimodal distribution, Patrick et al. stated that the median age for *Cronobacter* disease is 59 years, a number that has been widely quoted by the lay press and formula manufacturers’ representatives. As the authors surely knew, a median misrepresents a bimodal distribution. In the case of *Cronobacter*, it implies that middle-aged people are at risk of *Cronobacter* infection. That is precisely not the case – but it is a “statistic” which the CDC authors provided in their article and which PIF manufacturers’ lawyers can now use as evidence that late-middle-aged adults may transmit *Cronobacter* to their grandchildren.

*Cronobacter* infections are rare in both infants and adults but the similarity stops there. An equally egregious flaw in this often-cited article is that it obfuscates at least three distinct differences between *Cronobacter* infections in children and *Cronobacter* infections in adults. First, evidence increasingly supports that they are caused by different *Cronobacter* species and strains and the sources of these strains are different. Evidence to date strongly indicates that PIF is the primary source of invasive infections in young infants. The sources of infection/colonization in adults are unknown but these infections are likely transmitted nosocomially (i.e., through exposures related to their hospitalization and hospital care) and/or through contaminated nutrition these adults are receiving. Second, *Cronobacter* infections occur in previously healthy infants. Virtually all *Cronobacter* infection and colonization of adults occur in immunocompromised, elderly, and/or hospitalized adults who are ill with more significant infections and/or illnesses. Third, in infants, *Cronobacter* can cause meningitis, sepsis, and death. In adults, *Cronobacter* is an opportunistic organism that merely colonizes the patient or causes symptoms that are insignificant in comparison to that adult’s underlying medical problem and other infections. The authors compare apples to oranges when they state, “*the highest rates occurred among persons* ≥*80 years of age, followed by persons 70–79 years of age and infants*” (49).

**Figure 2:**
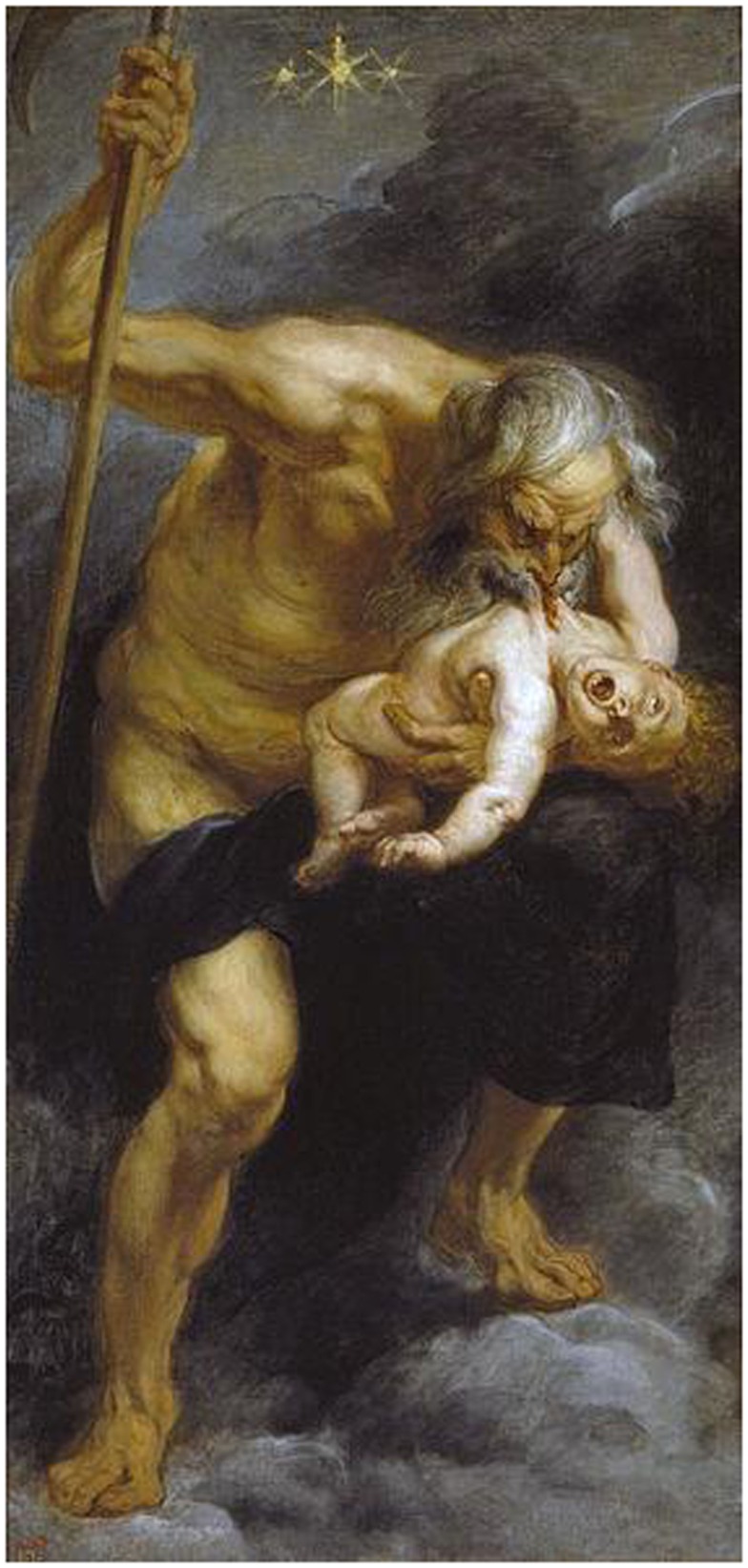
**Peter Paul Rubens’ rendering of Cronus devouring his son, Poseidon**.

Thanks to this article and CDC’s related website verbiage (51), the press, other authors, and public health agencies now parrot the phrase “*Cronobacter* infection occurs more frequently in adults than children.” This ignores the elephant-in-the-room, an elephant that is reflected in the very name of the genus: i.e., *Cronobacter* was named after Cronos, the Greek god who devoured his own children (Figure 2). Patrick et al.’s final recommendation concerning *Cronobacter* disease in adults is that “*routine, systematic surveillance and special studies will be essential for understanding these findings, identifying reservoirs of infection and vehicles of transmission, and developing effective prevention and control measures.” Cronobacter* is a rare infection; realistically, special studies would take decades and be expensive at a time when research dollars are scarce.

Rather than start from scratch, it would behoove CDC to identify and consolidate the many *Cronobacter* isolates it has obtained over the last 60+ years. One would expect that these isolates and their associated epidemiologic data have been safely stored. Patrick’s stated goals would be better and more efficiently served by testing those isolates using Baldwin’s MLST scheme and Tall’s pan genomic DNA microarray platform and analyzing the results.

U.S. Centers for Disease Control and Prevention appears to have thus far ignored the recent *Cronobacter* research related to strain typing and contamination distribution in PIF products. What, then, has been the response of other health agencies? This Frontiers Topic editorial review will end with an examination of the related current policies of three institutions: the FDA, the U.S. Special Supplemental Nutrition Program for Women, Infants, and Children (WIC), and the WHO.

By legal mandate, the FDA must oversee manufacturers of infant formulas and help ensure that these products are safe and support healthy growth in infants who consume them. On June 9, 2014 it finalized a rule that set standards for manufacturers of infant formula. Manufacturers had to comply with that rule as of September 8, 2014 (47). The new rules have, in general, eased FDA oversight of PIF manufacturing but the FDA has updated its protocol for testing end-products, including changing its statistical sampling plan to be in accordance with Dr. Jongenburger’s recommendations (44). The FDA notes that, with this sampling plan, “*when the production aggregate (*FDA’s new term for a lot or batch) *is sampled and the composite is tested, if the pathogen is not detected, the manufacturer has a 95% level of confidence that there would be* <*1 CFU Cronobacter spp. in 100 g powder*” (52). That, of course, continues to ignore the practical significance of *Cronobacter*’s clumping. Also, it merits noting that a 95% level of confidence means that, assuming the confidence interval/hypothesis test is one-sided, there is up to approximately a 5% chance of a type I error, i.e., that a production aggregate testing negative with FDA’s proposed testing scheme might actually contain ≥1 CFU *Cronobacter* spp. in 100 g powder but be released to market. This probability is indeed very low but since thousands of production aggregates are released to market each year, this risk is not inconsequential. If a released aggregate contains one or more contaminating clumps or clusters of a strain or sequence type that can produce invasive infection (e.g., ST4 or a virulent serogroup) and these clumps are fed to a young, susceptible infant, the consequences can be devastating.

Despite data to the contrary, on its “Consumer Update Page,” the FDA places RTF third on its list of three formula options and provides the following description of that option: “*Ready-to-feed* – *the most expensive form of formula that requires no mixing*” (47). Following the 2011 *Cronobacter* cases, the WIC Program reviewed its policies and decided to continue to preferentially support PIF over RTF, even in the first 2 months of life. WIC provides Federal grants to States for supplemental foods for approximately half of all U.S. infants. Parents on WIC cannot comparison shop; they must take what WIC provides them. WIC purchases infant formula at a discount, through a state-by-state exclusive contract bidding process, and provides that formula to non-breastfeeding or breast milk-supplementing mothers. WIC provides PIF to these mothers (53) and WIC programs instructs physicians to prescribe RTF only under certain, specific conditions, for their clients on WIC [e.g., Ref. (54)^5^].

The World Health Organization’s current recommendations related to *Cronobacter* are that PIF be reconstituted with water that has been boiled and then cooled to 70°C, to inactivate any *Cronobacter* contaminating the PIF (55, 56). There are at least three problems with this recommendation, one of which was highlighted in Dr. Parra Flores’ Frontiers Topic manuscript (35). First, it is unlikely that this recommendation will be accurately followed in a home setting. Few caretakers will routinely measure the temperature of boiled water before they mix it with PIF. The time required will vary with ambient temperature. If the water is cooled too long, the previously boiled, now warm water will incubate contaminating *Cronobacter*, not kill it. Second, some organizations disagree with this recommendation because of concerns that it may destroy heat-sensitive nutrients, change some formulas’ physical characteristics, cannot be accurately followed in a real-world setting, and/or because the hot water could injure personnel preparing the formula (21). Third, the rehydration instructions on some PIF labels do not comply with WHO guidelines.

## Conclusion

In an ideal world, medical, laboratory, and epidemiologic researchers and health agencies work hand-in-hand to further scientific knowledge and apply that knowledge to benefit society. In the past, in regard to *Cronobacter*, this interaction was more or less ideal and led to significant progress. In recent years, laboratorians have been doing more than their fair share. It is time for epidemiologists and health agencies to view this issue with fresh eyes and step up to the plate.

## Author Contributions

JJ was responsible for the concept, literature review, and manuscript preparation.

## Conflict of Interest Statement

The author has not received any funding for this study. She has acted as an expert witness in legal cases related to *Cronobacter* infection; therefore, if this study leads to preventive action, it is, in a practical sense, contrary to her personal financial interests.

## Footnotes

^1^ftp://ftp.fao.org/docrep/fao/009/a0707e/a0707e00.pdf and http://www.fstools.org/esakmodel/ESAKRAModelWizard.aspx

^2^http://ec.europa.eu/food/food/rapidalert/reports/week8-2009_en.pdf

^3^http://pubmlst.org/Cronobacter/

^4^http://www.cdc.gov/cronobacter/investigation.html#advice

^5^https://www1.maine.gov/dhhs/mecdc/health-equity/wic/documents/health-RTFricestarchformulas.pdf
